# The prevalence and influencing factors in anxiety in medical workers fighting COVID-19 in China: a cross-sectional survey

**DOI:** 10.1017/S0950268820001107

**Published:** 2020-05-20

**Authors:** Chen-Yun Liu, Yun-zhi Yang, Xiao-Ming Zhang, Xinying Xu, Qing-Li Dou, Wen-Wu Zhang, Andy S. K. Cheng

**Affiliations:** 1 Department of Emergency, The Affiliated Baoan Hospital of Southern Medical University, People's Hospital of Shenzhen Baoan District, Shenzhen, China; 2 Department of Nursing, The Affiliated Baoan Hospital of Southern Medical University, People's Hospital of Shenzhen Baoan District, Shenzhen, China; 3 Department of Rehabilitation Sciences, The Hong Kong Polytechnic University, Hong Kong, China

**Keywords:** Anxiety, COVID-19, medical workers

## Abstract

The coronavirus disease 2019 (COVID-19) outbreak caused by the severe acute respiratory syndrome coronavirus 2 (SARS-Cov-2 virus) has been sustained in China since December 2019, and has become a pandemic. The mental health of frontline medical staff is a concern. In this study, we aimed to identify the factors influencing medical worker anxiety in China during the COVID-19 outbreak. We conducted a cross-sectional study to estimate the prevalence of anxiety among medical staff in China from 10 February 2020 to 20 February 2020 using the Zung Self-rating Anxiety Scale (SAS) to assess anxiety, with the criteria of normal (⩽49), mild (50–59), moderate (60–70) and severe anxiety (⩾70). We used multivariable linear regression to determine the factors (e.g. having direct contact when treating infected patients, being a medical staff worker from Hubei province, being a suspect case) for anxiety. We also used adjusted models to confirm independent factors for anxiety after adjusting for gender, age, education and marital status. Of 512 medical staff in China, 164 (32.03%) had had direct contact treating infected patients. The prevalence of anxiety was 12.5%, with 53 workers suffering from mild (10.35%), seven workers suffering from moderate (1.36%) and four workers suffering from severe anxiety (0.78%). After adjusting for sociodemographic characteristics (gender, age, education and marital status), medical staff who had had direct contact treating infected patients experienced higher anxiety scores than those who had not had direct contact (*β* value = 2.33, confidence interval (CI) 0.65–4.00; *P* = 0.0068). A similar trend was observed in medical staff from Hubei province, compared with those from other parts of China (*β* value = 3.67, CI 1.44–5.89; *P* = 0.0013). The most important variable was suspect cases with high anxiety scores, compared to non-suspect cases (*β* value = 4.44, CI 1.55–7.33; *P* = 0.0028). In this survey of hospital medical workers during the COVID-19 outbreak in China, we found that study participants experienced anxiety symptoms, especially those who had direct clinical contact with infected patients; as did those in the worst affected areas, including Hubei province; and those who were suspect cases. Governments and healthcare authorities should proactively implement appropriate psychological intervention programmes, to prevent, alleviate or treat increased anxiety.

## Introduction

The coronavirus disease 2019 (COVID-19) outbreak caused by severe acute respiratory syndrome coronavirus 2 (SARS-Cov-2) emerged in Wuhan, China, spread to the entire country from the end of December 2019, and has attracted enormous concern from around the world [1]. In March 2020, the World Health Organization (WHO) declared COVID-19 a pandemic. It is reported that the number of infected patients is more than 3 024 059, with 208 112 deaths worldwide as of 29 April 2020 (http://www.who.int). This makes COVID-19 more serious than SARS, a similar epidemic disease [2]. The Chinese government has implemented numerous measures, including quarantines, reducing the use of public transportation and temporarily cancelling work and school, to control this disease [3]. In addition, government authorities sent about 30 000 medical staff from each Chinese province to fight COVID-19. COVID-19 is characterised by complexity, including human-to-human transmission, asymptomatic carrier transmission and high transmission efficiency, which has led to a worldwide pandemic [4–6]. Medical staff are frontline workers who treat infected patients, however, with a higher risk of exposure to themselves. Current source data have presented the proportion of infected medical staff at 3.8%, mainly due to early non-protected contact with infected patients [7, 8]. Several previous studies reported that medical staff might suffer adverse psychological disorders, such as anxiety, fear and stigmatisation, which occurred during the SARS and Ebola outbreaks, and could exert an adverse effect on care quality [9–12]. Medical staff must wear heavy protective garments and an N95 mask, making it much more difficult to carry out medical operations or procedures than under normal conditions. These factors, together with the fear of being contagious and infecting others, could increase the possibility of psychological issues among medical staff. Koh *et al*. found that more than half of the clinical staff reported increased work stress (56%) and workload (53%) during the SARS epidemic in Singapore [13]. In addition, a Hong Kong study found that health workers suffered high anxiety scores after directly treating confirmed SARS patients [14]. Therefore, it is very important to study medical workers' mental health status. This outbreak has highlighted the fragility of mental resilience [15]. Studies exploring the prevalence of anxiety among medical staff during the COVID-19 outbreak in China are limited. Our study's aim was to examine the anxiety levels of frontline healthcare workers and to identify the risk factors for anxiety in China during the COVID-19 epidemic. Our findings might help governments or health authorities to recognise the causes of increased anxiety in healthcare workers, and then to provide early effective measures to reduce that anxiety.

## Methods

This is a descriptive quantitative cross-sectional study that was used to explore the prevalence and factors linked to anxiety in frontline medical staff. Data were collected from 10 February 2020 to 20 February 2020 in China during the COVID-19 epidemic. Informed consent was provided by subjects before study commencement. After that, we distributed self-report questionnaires to healthcare workers via WeChat.

### Study participants

Participating healthcare staff included doctors, nurses and administrative workers at hospitals equipped with a fever clinic or a COVID-19 ward in different regions in China. Administrative staff work in administration and did not directly engage in the treatment or care of infected patients.

### Materials

The questionnaire consisted of three sections: (1) Demographic characteristics, such as gender, age, marital status, level of education, hospital department and city. (2) Questions included the following: e.g. Have you ever directly treated a patient with COVID-19? Have you been to Hubei province in the last month? Are you a suspect case who had direct contact with a confirmed case or do you have fever, fatigue, cough? Did you adhere to the preventive and control measures in your community? Do you need psychological treatment? (3) The Zung Self-rating Anxiety Scale (SAS) [16] was used to assess anxiety levels in medical staff. In SAS, there are 20 items ranked on a 1–4 scale, with total raw scores ranging from 20 to 80. In addition, published studies conducted in China have reported satisfactory reliability and validity [17]. The higher the score, the higher the degree of anxiety. The SAS scores were classified into four categories, including normal (⩽49), mild anxiety (50–59), moderate anxiety (60–70) and severe anxiety (⩾70), after standardizing the score based on raw data multiplied by 1.25. Previous studies have shown that SAS internal consistency reliability was 0.66–0.80 and the Cronbach's *α* was 0.87 [18].

### Ethical considerations

Ethical approval from the ethics committee of the People's Hospital of Baoan District, Shenzhen (Certificate: BYL20200202) was obtained, with written consent provided by all participants. Medical staff whose SAS scores were more than 60 points were informed of their score and a researcher contacted them to ask whether they wanted psychological treatment. If they accepted, our team helped them make an online appointment for a psychological consultation.

### Statistical data analyses

Continuous and categorical variates were summarised as mean values ± standard deviation (s.d.) and frequency (percentage), respectively. We used the *χ*^2^ test to identify the differences in categorical variables between groups, and the Student's *t* test was used to determine the differences in continuous variables between groups. If the data showed a skewed distribution, the Kruskal–Wallis *H* test was used. In addition, a univariate analysis model was used to identify the relationship between risk factors and anxiety score. Finally, our paper also lists the unadjusted and adjusted multivariate linear regression analysis model. Statistically significant differences were identified as a two-sided *P* value <0.05. All analyses were conducted using EmpowerStats (http://www.empowerstats.com, X&Y Solutions, Inc., Boston, MA, USA) and software package R (http://www.r-project.org).

## Results

Patient baseline characteristics are presented in Table 1.

**Table 1.** Participant demographic data

Variables	Mean + s.d, or *N*,%
Anxiety score	39.56 ± 8.91
Gender	*N* (%)
Female	433 (84.57%)
Male	79 (15.43%)
Age group	
18–39	386 (75.39%)
40–59	125 (24.41%)
⩾60	1 (0.20%)
Education	
Middle school or lower	2 (0.39%)
High school or higher	24 (4.69%)
College or higher	486 (94.92%)
Marital status	
Single	185 (36.13%)
Married	320 (62.50%)
Divorced	7 (1.37%)
Hubei province	
No	439 (85.74%)
Yes	73 (14.26%)
Location	
Countryside	8 (1.56%)
City	504 (98.44%)
Suspect cases	
No	471 (91.99%)
Yes	41 (8.01%)
Department	
Clinical department	369 (72.07%)
Fever clinic	68 (13.28%)
Service/managerial	75 (14.65%)
Have direct treatment	
No	348 (67.97%)
Yes	164 (32.03%)
Satisfaction with community prevention	
No	468 (91.41%)
Yes	44 (8.59%)
Need psychological treatment	
No	463 (90.43%)
Yes	49 (9.57%)
Anxiety	
Normal	448 (87.5%)
Mild	53 (10.35%)
Moderate	7 (1.36%)
Severe	4 (0.78%)

A total of 600 medical staff was surveyed, with 512 subjects completing the questionnaire and a dropout rate of 14.6%. The majority of the sample was female (84.57%) and the largest age group was 18–39 years old. Most sample participants were married (62.50%) and the majority of subjects had a tertiary level of education. Most health workers came from a clinical department (72.07%), with 13.28% setting up clinics to screen patients for fever, and 14.65% involved in administration. A total of 164 health workers had directly treated confirmed patients as shown in Table 1, with 14.26% of respondents coming from Hubei province, the most severely affected area. A total of 53 respondents suffered from mild anxiety (10.35%), seven from moderate anxiety (1.36%) and four from severe anxiety (0.78%).

### Comparing the differences between two groups (direct treatment *vs.* non-direct treatment)

Table 2 shows the differences between medical staff who had directly treated patients with a confirmed case of COVID-19 and those who had not. The average anxiety score was significantly higher in medical staff who had directly treated confirmed cases, compared with those who had not (41.11 ± 9.79 *vs.* 38.83 ± 8.38, *P* = 0.007). There is a significant difference between these two groups in terms of the variables of gender, department, Hubei province, suspect case, satisfaction with the effectiveness of community prevention measures and need for psychological counselling. However, the variables of age group, education, marital status and location were not obvious in these two groups.

**Table 2.** Comparing the differences between two groups (direct treatment *vs.* non-direct treatment)

Variables	Group 1 (direct contact treatment)	Group 2 (non-direct contact treatment)	Standardise diff.	*P* value
*N*	348	164		
Anxiety score	38.83 ± 8.38	41.11 ± 9.79	0.25 (0.06–0.44)	0.007
Gender			0.26 (0.07–0.44)	0.005
Female	305 (87.64%)	128 (78.05%)		
Male	43 (12.36%)	36 (21.95%)		
Age group			0.13 (−0.06 to 0.31)	0.283
18–39	260 (74.71%)	126 (76.83%)		
40–59	88 (25.29%)	37 (22.56%)		
⩾60	0 (0.00%)	1 (0.61%)		
Education			0.17 (−0.01 to 0.36)	0.223
Middle school or lower	1 (0.29%)	1 (0.61%)		
High school or higher	20 (5.75%)	4 (2.44%)		
College or higher	327 (93.97%)	159 (96.95%)		
Marital status			0.11 (−0.08 to 0.29)	0.524
Single	120 (34.48%)	65 (39.63%)		
Married	223 (64.08%)	97 (59.15%)		
Divorced	5 (1.44%)	2 (1.22%)		
Department			0.90 (0.70–1.09)	<0.001
Clinical department	255 (73.28%)	114 (69.51%)		
Fever clinic	20 (5.75%)	48 (29.27%)		
Administration	73 (20.98%)	2 (1.22%)		
Hubei province			0.92 (0.73–1.12)	<0.001
No	336 (96.55%)	103 (62.80%)		
Yes	12 (3.45%)	61 (37.20%)		
Location			0.04 (−0.14 to 0.23)	0.667
Countryside	6 (1.72%)	2 (1.22%)		
City	342 (98.28%)	162 (98.78%)		
Suspect case			0.65 (0.46–0.84)	<0.001
No	342 (98.28%)	129 (78.66%)		
Yes	6 (1.72%)	35 (21.34%)		
Satisfaction with community prevention			0.24 (0.05–0.43)	0.008
No	326 (93.68%)	142 (86.59%)		
Yes	22 (6.32%)	22 (13.41%)		
Need psychological treatment			0.21 (0.03–0.40)	0.019
No	322 (92.53%)	141 (85.98%)		
Yes	26 (7.47%)	23 (14.02%)		

### Univariate analysis

Table 3 shows the risk factors associated with increased anxiety scores using univariate analysis.

**Table 3.** Risk factors associated with increased anxiety scores by using univariate analysis

Variables	Frequency	Statistics	Anxiety score
Gender			
Female	433 (84.57%)	39.23 ± 8.63	Reference
Male	79 (15.43%)	39.40 ± 9.07	0.36 (−1.78 to 2.50) 0.7436
Age group			
18–39	386 (75.39%)	39.39 ± 8.34	Reference
40–59	125 (24.41%)	40.11 ± 10.49	0.72 (−1.08 to 2.52) 0.4339
⩾60	1 (0.20%)	33.75 ± NA	−5.64 (−23.14 to 11.86) 0.5278
Education			
Middle school or lower	2 (0.39%)	38.75 ± 8.83	Reference
High school or higher	24 (4.69%)	39.11 ± 8.44	0.36 (−12.51 to 13.24) 0.9558
College or higher	486 (94.92%)	39.58 ± 8.94	0.83 (−11.57 to 13.23) 0.8956
Marital status			
Single	185 (36.13%)	39.55 ± 7.83	Reference
Married	320 (62.50%)	39.55 ± 9.45	0.00 (−1.62 to 1.62) 0.9994
Divorced	7 (1.37%)	39.64 ± 11.05	0.09 (−6.65 to 6.83) 0.9794
Department			
Clinical department	369 (72.07%)	39.65 ± 8.78	Reference
Fever clinic	68 (13.28%)	40.93 ± 9.8	1.28 (−1.02 to 3.58) 0.2757
Administration	75 (14.65%)	37.8 ± 8.49	−1.86 (−4.06 to 0.35) 0.0995
Direct treatment			
No	348 (67.97%)	38.77 ± 8.04	Reference
Yes	164 (32.03%)	41.16 ± 10.77	2.28 (0.64–3.92) 0.0068
Hubei province			
No	439 (85.74%)	38.91 ± 8.42	Reference
Yes	73 (14.26%)	42.13 ± 10.51	3.71 (1.53–5.90) 0.0009
Location			
Countryside	8 (1.56%)	39.45 ± 9.47	Reference
City	504 (98.44%)	39.25 ± 8.65	−1.09 (−7.31 to 5.14) 0.7326
Suspect cases			
No	471 (91.99%)	39.05 ± 8.62	Reference
Yes	41 (8.01%)	42.52 ± 9.69	4.13 (1.30–6.95) 0.0043
Satisfaction with community prevention			
No	468 (91.41%)	39.05 ± 8.60	Reference
Yes	44 (8.59%)	40.95 ± 9.52	2.60 (−0.15 to 5.35) 0.0642
Need psychological treatment			
No	463 (90.43%)	39.17 ± 8.75	Reference
Yes	49 (9.57%)	40.05 ± 8.49	2.33 (−0.29 to 4.94) 0.0824

The results found that direct treatment, residence in Hubei province and suspect cases were associated with increased anxiety scores (*P* ⩽ 0.05). However, we found that the variables of gender, age, education, marital status, location, healthcare workers' satisfaction with the effectiveness of community prevention measures and the need for psychological counselling did not increase anxiety scores (all *P* ⩾ 0.05).

### Multivariable analysis to evaluate the independent impact of direct treatment on anxiety scores among medical workers using non-adjusted and adjusted linear regression analysis

We used multivariable linear regression to detect the relationship between direct treatment and anxiety score using a different model. The results show that direct treatment is an independent risk factor for an increased anxiety score (*β* value = 2.280, 95% confidence interval (CI) 0.636–3.924; *P* = 0.0068) in an unadjusted model, together with suspect cases (*β* value = 4.13, 95% CI 1.30–6.95; *P* = 0.0043), and being a medical worker from Hubei province (*β* value = 3.71, 95% CI 1.53–5.90; *P* = 0.0009). In addition, all of these associations were still significant after adjusting for the variables of gender, age, education and marital status, with detailed results shown in Table 4.

**Table 4.** Multiple linear regression for anxiety score

Variables (exposure)	Non-adjusted (*β*, 95% CI, *P)*	Adjusted (*β*, 95% CI, *P)*
Direct contact treatment		
No	Reference	Reference
Yes	2.28 (0.64–3.92) 0.0068	2.33 (0.65–4.00) 0.0068
Suspect cases		
No	Reference	Reference
Yes	4.13 (1.30–6.95) 0.0043	4.44 (1.55–7.33) 0.0028
Hubei province		
No	Reference	Reference
Yes	3.71 (1.53–5.90) 0.0009	3.67 (1.44–5.89) 0.0013

Outcome: anxiety score.

Non-adjusted model adjusted for: we did not adjust for other covariants.

Adjusted model adjusted for: gender, age, education, marital status.

Ref: reference.

## Discussion

Our study examined the prevalence of anxiety in medical staff and identified their risk factors for increased anxiety. Our results found that working in COVID-19 patient care, suspect cases and living in Hubei province were all risk factors for increased anxiety scores, which is an important finding suggesting that government authorities need to implement measures to alleviate mental health symptoms in healthcare workers early on. New bio-disasters – including SARS, Ebola, H1N1, Middle East respiratory syndrome (MERS) and the novel coronavirus – are profoundly associated with adverse psychological effects on medical staff, including depression, anxiety and insomnia. Using SAS, we found that 12.5% of medical staff experienced anxiety, which is less than in a recent study by Lai *et al*., in which 44.6% of respondents reported anxiety symptoms during the COVID-19 outbreak [19]. The main possible reasons were the different tool assessments and different stages of COVID-19. By the time our study was conducted, nearly 4 weeks after the first COVID-19 patient was confirmed in Wuhan, China at the end of December 2019, healthcare workers had apparently had sufficient time to adjust to caring for infected patients and to feel confident in doing so. In addition, government and healthcare authorities were providing stricter and safer protective measures to support them, which to some extent reduced worker anxiety, in contrast to the early peak of the epidemic. However, a study by Jeong *et al*. reported the prevalence of anxiety symptoms in the general population who were not diagnosed with MERS and required 2 weeks of isolation was 7.6% (95% CI 6.3–8.9%), which is less than in our study [20]. The discrepancy might be due to the fact that there were only 267 health workers (16.1%), and the authors used the seven-item Generalized Anxiety Disorder Scale to assess anxiety, with a cut-off of 5 points confirming mild anxiety. Nevertheless, medical workers who provided direct treatment or care for infected patients suffered higher anxiety scores, compared to those who were not caring for COVID-19 patients. Previous studies have reported that psychological symptoms, such as anxiety, depend on the epidemic phase [21]. This is because medical workers might have been able to adapt psychologically, after gradually learning more about SARS and obtaining rich clinical experience in the treatment and care of infected patients.

The results show that health workers from Hubei, the most severely affected area, had higher anxiety scores (*β* value = 3.71, CI 1.53–5.90; *P* = 0.0009) compared to health workers from other regions. Staff working in hospitals in Hubei suffered heavy workloads due to the increasing number of infected cases requiring centralisation to designated hospitals for standard isolation treatment. Additionally, the media have reported that medically protective materials, such as N95 masks, goggles and protective clothing, were severely deficient during the early stages of the outbreak [22]. All of these factors invisibly aggravated the psychological burden.

In addition, our results found that staff with suspect infection cases had higher anxiety scores than non-suspect cases (*β* value = 4.44, CI 1.55–7.33; *P* *=* 0.0028). Wu *et al.* [11] conducted a survey on the psychological problems of hospital employees during the SARS epidemic, and found that respondents who were quarantined had higher levels of post-traumatic stress than those without exposure. Medical staff became suspect cases mainly through hospital-related transmission. Suspect cases with a high risk of infection were required to remain isolated for 2 weeks under medical observation [23]. During that time, they might have suffered a dilemma: on the one hand, most people fear becoming infected with SARS-CoV-2, while on the other hand, they struggle with not taking on responsibility for fighting COVID-19. They also worry about the health risks to their own family, leading to a psychological burden. This complex situation could further intensify anxiety in medical staff. Therefore, governments should focus on potential psychological problems among suspect cases in medical staff, and provide effective mental health measures to alleviate suffering.

After adjusting the confounding factors, providing direct treatment to infected patients was an independent factor in increased anxiety scores, compared to not providing direct treatment to COVID-19 patients. Our finding is in line with a recent study conducted on healthcare workers at the onset of the infection outbreak [19]. This study found that healthcare workers who directly diagnosed, treated or looked after patients with COVID-19 were more stressed and psychologically impacted than workers who did not have direct contact with COVID cases. Participating in treatments or procedures for infected patients was challenging for frontline staff, who experienced stress, as they were at high potential risk of infection due to the illness' characteristics of high transmission efficiency, rapid deterioration and pathogenicity. Hence, healthcare workers, such as those having direct contact with patients, suffered higher anxiety symptom scores than staff who were at low risk. Therefore, although the Chinese government and society compliment medical personnel for their dedication in fighting COVID-19 – which could make medical workers feel honoured and proud to participate in this difficult mission – authorities should also focus on implementing measures to target workers' mental health. As the SARS-Cov-2 epidemic is a global issue, fighting COVID-19 appears to be a sustained event, which might result in medical personnel suffering psychological problems.

Our study has some limitations. First, the questionnaires were distributed non-randomly via WeChat, so a selective bias exists in our study. Additionally, the number of medical staff from Hubei was in a minority (14.26%), which means that our study does not completely reflect the entire mental health picture of Chinese medical staff in quarantine. Second, we did not investigate other important outcomes, such as insomnia, depression and post-traumatic stress related to the functioning of healthcare workers. Therefore, we cannot provide information about how mild anxiety has any impact on the function of medical workers. It would be worthwhile to study these possible factors in future research. Third, we used the SAS, which is widely applied in China, but is an old anxiety scale now rarely used in Western countries. In order to keep the questionnaire at a reasonable length and to promote an acceptable response rate, we did not use the Chinese Classification of Mental Disorders (CCMD), International Classification of Diseases and Related Health Problems (ICD-11) or Diagnostic and Statistical Manual of Mental Disorders 5th edition (DSM-5 scale) to evaluate anxiety, which could possibly have jeopardised the generalisation of our study and limited its effectiveness in providing more information for other countries around the world. Fourth, COVID-19-related anxiety is in fact likely to be dynamic, waxing and waning in response to stressors. Medical workers' anxiety may spike, for example, when there is a surge of patients into their hospital, and abate when the influx of cases abates. Wang *et al*. [24] reported a study that explored the immediate psychological responses during the initial stage of the COVID-19 epidemic in China and found the prevalence of anxiety (28.8% reported moderate to severe anxiety symptoms) was higher than in our study, since their study period was at the peak of the COVID-19 outbreak in China, with similar results reported by Lai *et al*. (44.6% had anxiety symptoms). In our study, however, we were unable to explore anxiety in healthcare workers at a different time; therefore, we cannot compare our results to the different stages of anxiety among healthcare workers. Fifth, our study used a cross-sectional design that cannot determine causality for factors and outcome. In addition, a comprehensive assessment, including demographic factors, such as years of experience, having children or not and a history of mental disorders, would be beneficial in analysing potential anxiety factors. Finally, we were unable to distinguish pre-existing anxiety *vs.* new cases of anxiety. However, our study also has some strengths. To our knowledge, this is the first study to assess anxiety levels among medical staff in China during the period of COVID-19, and we used comprehensive statistical data analyses to ensure our results are reliable. In addition, our findings have the potential to encourage policymakers and governments to consider offering early interventions to alleviate possible mental health problems in medical workers.

## Conclusion

Our study found the prevalence of anxiety is mild; however, medical staff who had had direct contact through treatment of infected patients may experience an increase in their anxiety scores, compared to workers having no direct contact with infected patients. In addition, healthcare workers who were quarantined in Hubei province and those who were suspect cases also saw increased anxiety scores. Therefore, governments and healthcare authorities should proactively implement appropriate measures, such as providing psychological counselling services, to prevent, alleviate or treat increased anxiety among medical staff, especially in the worst affected areas, during the COVID-19 pandemic.
